# Evolution and improved outcomes in the era of multimodality treatment for extended pancreatectomy

**DOI:** 10.1093/bjsopen/zrae065

**Published:** 2024-08-01

**Authors:** Vikram A Chaudhari, Aditya R Kunte, Amit N Chopde, Vikas Ostwal, Anant Ramaswamy, Reena Engineer, Prabhat Bhargava, Munita Bal, Nitin Shetty, Suyash Kulkarni, Shraddha Patkar, Manish S Bhandare, Shailesh V Shrikhande

**Affiliations:** GI & HPB Surgical Services, Department of Surgical Oncology, Tata Memorial Centre, Homi Bhabha National Institute, Mumbai, India; GI & HPB Surgical Services, Department of Surgical Oncology, Tata Memorial Centre, Homi Bhabha National Institute, Mumbai, India; GI & HPB Surgical Services, Department of Surgical Oncology, Tata Memorial Centre, Homi Bhabha National Institute, Mumbai, India; Department of Medical Oncology, Tata Memorial Centre, Homi Bhabha National Institute, Mumbai, India; Department of Medical Oncology, Tata Memorial Centre, Homi Bhabha National Institute, Mumbai, India; Department of Radiation Oncology, Tata Memorial Centre, Homi Bhabha National Institute, Mumbai, India; Department of Medical Oncology, Tata Memorial Centre, Homi Bhabha National Institute, Mumbai, India; Department of Pathology, Tata Memorial Centre, Homi Bhabha National Institute, Mumbai, India; Department of Interventional Radiology, Tata Memorial Centre, Homi Bhabha National Institute, Mumbai, India; Department of Interventional Radiology, Tata Memorial Centre, Homi Bhabha National Institute, Mumbai, India; GI & HPB Surgical Services, Department of Surgical Oncology, Tata Memorial Centre, Homi Bhabha National Institute, Mumbai, India; GI & HPB Surgical Services, Department of Surgical Oncology, Tata Memorial Centre, Homi Bhabha National Institute, Mumbai, India; GI & HPB Surgical Services, Department of Surgical Oncology, Tata Memorial Centre, Homi Bhabha National Institute, Mumbai, India

## Abstract

**Background:**

The evolution and outcomes of extended pancreatectomies at a single institute over 15 years are presented in this study.

**Methods:**

A retrospective analysis of the institutional database was performed from 2015 to 2022 (period B). Patients undergoing extended pancreatic resections, as defined by the International Study Group for Pancreatic Surgery, were included. Perioperative and survival outcomes were compared with data from 2007–2015 (period A). Regression analyses were used to identify factors affecting postoperative and long-term survival outcomes.

**Results:**

A total of 197 (16.1%) patients underwent an extended resection in period B compared to 63 (9.2%) in period A. Higher proportions of borderline resectable (5 (18.5%) *versus* 51 (47.7%), *P* = 0.011) and locally advanced tumours (1 (3.7%) *versus* 24 (22.4%), *P* < 0.001) were resected in period B with more frequent use of neoadjuvant therapy (6 (22.2%) *versus* 79 (73.8%), *P* < 0.001). Perioperative mortality (4 (6.0%) *versus* 12 (6.1%), *P* = 0.81) and morbidity (23 (36.5%) *versus* 83 (42.1%), *P* = 0.57) rates were comparable. The overall survival for patients with pancreatic adenocarcinoma was similar in both periods (17.5 (95% c.i. 6.77 to 28.22) *versus* 18.3 (95% c.i. 7.91 to 28.68) months, *P* = 0.958). Resectable, node-positive tumours had a longer disease-free survival (DFS) in period B (5.81 (95% c.i. 1.73 to 9.89) *versus* 14.03 (95% c.i. 5.7 to 22.35) months, *P* = 0.018).

**Conclusion:**

Increasingly complex pancreatic resections were performed with consistent perioperative outcomes and improved DFS compared to the earlier period. A graduated approach to escalating surgical complexity, multimodality treatment, and judicious patient selection enables the resection of advanced pancreatic tumours.

## Teaser

Outcomes of extended pancreatic resections performed from 2015 to 2022 at the Tata Memorial Centre, Mumbai were compared to previously published data from an earlier time period in this retrospective analysis. An increased use of neoadjuvant therapy along with an increased complexity of surgical resection was noted. Surgical outcomes were comparable despite increased complexity. Disease-free survival of node-positive resectable pancreatic cancers was improved.

## Introduction

Complete surgical resection is the most critical predictor in achieving long-term survival in localized pancreatic adenocarcinoma (PDAC)^1–5^. A majority of PDACs are either borderline resectable (BR) or locally advanced (LA) due to frequent vascular involvement, posing a challenge for a margin-negative resection. From its initial description by Fortner in 1973^6^, portal vein resections have now become a standard of care for the surgical resection of advanced pancreatic cancers^7–10^. With improved surgical techniques and more effective systemic therapy^11,12^, more aggressive resections involving major arteries have been made possible, leading to improved survival in a select group of patients^13–20^. Pancreatic resections involving additional resection of vascular structures and adjacent organs are associated with higher perioperative mortality and morbidity compared to standard pancreatic resections^21^. Hartwig *et al*. streamlined the definition of extended pancreatic resections in a consensus statement of the international study group of pancreatic surgery (ISGPS) in 2014^22^. A previous study of 63 cases of extended pancreatic resections was performed over 9 years (2007–2015), and it found a postoperative mortality rate of 6% and a major morbidity rate of 67%^21^.

The practice at Tata Memorial Centre has evolved over the years in tandem with other high-volume pancreatic surgery centres worldwide. Over the last decade, it has seen progressively higher volumes, more complex resections, and increased utilization of neoadjuvant therapy (NAT). In the present study, the aim was to assess the impact of this evolution over time.

## Methods

### Study design

A prospectively maintained institutional database of pancreatic resections was analysed. Patients who underwent an extended pancreatic resection between 1 September 2015 and 31 August 2022 were included in the study. This period was designated as ‘period B’. Extended resections were defined by the ISGPS criteria^22^ as pancreatic resections with additional resection of adjacent organs, vascular resections, or extended lymph node dissection. Perioperative, pathologic and survival outcomes were assessed. Perioperative mortality, perioperative morbidity, ideal outcomes, failure-to-rescue rate, margin positive (R+) rate, DFS, and overall survival (OS) for period B were compared to previously published results from an earlier period (January 2006 to August 2015), which was designated as ‘period A’^21^.

Resectability criteria were assessed by the National Comprehensive Cancer Network (NCCN) guidelines^23^. Perioperative mortality included death from any cause within 90 days from the date of surgery. A complication classified as Clavien–Dindo grade IIIa or higher was defined as major morbidity^24^. Ideal outcomes for pancreatic surgery were defined by the absence of in-hospital mortality, major morbidity, postoperative pancreatic fistula (POPF) grade B or C, re-exploration, readmission and hospital stay > 75th percentile^25^. The 75th percentile of hospital stay was derived from the entire institutional cohort of pancreatic surgeries. Postoperative complications such as a POPF, biliary leak, post-pancreatectomy haemorrhage (PPH), chyle leak and delayed gastric emptying (DGE) were defined following the ISGPS criteria^26–29^. Surgical specimens were analysed by multiple pathologists and the margin status was reported by the standardized LEEDS pathology protocol^30^. Pathologic tumour response was reported according to the College of American Pathologists (CAP) protocol^31^.

Survival outcomes were analysed only for PDAC. DFS was the period from the date of surgery to the date of recurrence or the date of last follow-up. OS was the period from the date of diagnosis to death from any cause or the date of last follow-up. The failure-to-rescue rate was the ratio of patients who died within 90 days of surgery to those who suffered major morbidity, expressed as a percentage^32^.

### Treatment planning

Response assessment to chemotherapy was based on radiological, biochemical and patient-related subjective criteria. Borderline resectable pancreatic cancer (BRPC) patients with no or limited (<180 degrees with a single artery) arterial contact and a favourable response after four chemotherapy cycles were planned for surgical resection after an additional 2–4 chemotherapy cycles. BRPC with significant arterial contact (>180 degrees), contact with multiple arteries, and locally advanced pancreatic cancer (LAPC) patients were planned for a total neoadjuvant therapy (TNT), defined as completion of ≥ 6 cycles of neoadjuvant chemotherapy (NACT)^33^, with an aim to complete 10–12 cycles before surgery. LAPCs and BRPCs with arterial involvement were considered for stereotactic body radiotherapy (SBRT). The routine follow-up schedule consisted of in-person visits to the outpatient clinic every 3 months for the first 2 years after surgery and every 6 months for the following 3 years. ‘Recurrence’ was documented upon obtaining tissue diagnosis of suspicious lesions, unless the radiology and tumour marker profile was definitive enough to obviate the need for a tissue diagnosis.

### Statistical analysis

Categorical variables were expressed as proportions, whereas continuous variables were expressed as median and range. Categorical variables were compared using a Pearson Chi-square test, and continuous variables were compared using a Mann–Whitney/U-test. Factors impacting mortality and morbidity were analysed by a stepwise binomial logistic regression. Patient age, sex, ASA grade, BMI, tumour size, preoperative biliary stenting, preoperative albumin level, NAT, type of pancreatectomy, the complexity of surgery, number of additional organs resected, intraoperative blood loss and duration of surgery were the variables analysed in a stepwise multivariate regression to predict postoperative mortality (90-day) and morbidity. Receiver operating characteristic (ROC) curves were plotted for continuous variables identified as significant predictors of postoperative mortality, and the area under ROC (AUC) was analysed. The median duration of follow-up was assessed by using the reverse Kaplan–Meier method. Survival outcomes were plotted using Kaplan–Meier curves and were compared using a log-rank test. Factors impacting long-term survival were analysed by using a stepwise Cox regression. *P* ≤ 0.05 was considered statistically significant. This study was performed in accordance with ethical guidelines laid out in the Helsinki Declaration (2008) and after obtaining approval from the institutional ethics committee

## Results

### Patient characteristics

**Fig. 1:**
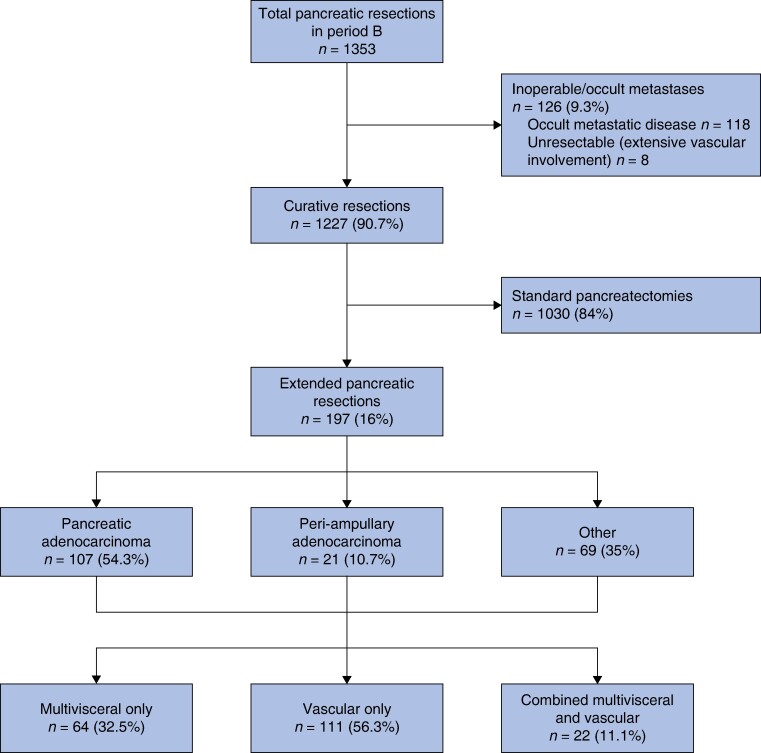
Consort diagram of the study population

A total of 1227 resections were performed in period B, of which 197 (16%) were extended pancreatic resections (*Fig. 1*). This was significantly higher as compared to 63/683 (9.2%) extended resections in period A (*P* < 0.001). Of the extended resections performed, 27 (42.9%) were PDACs in period A compared to 107 (54.3%) in period B (*P* = 0.116). The number of vascular resections increased marginally in period B (36 (57.1%) in period A *versus* 133 in period B (67.5%), *P* = 0.093) but arterial resections and divestment procedures were significantly higher (2 in period A (5.5%) *versus* 39 in period B (29.3%), *P* = 0.001).

Staging laparoscopy was used selectively in patients with elevated carbohydrate antigen (CA 19-9 levels). A superior mesenteric artery (SMA)-first approach was utilized in all cases. End-to-end reconstruction was the preferred venous or arterial reconstruction approach after segmental resection. A saphenous vein graft was used for arterial reconstruction if end-to-end reconstruction was not feasible. A polytetrafluoroethylene (PTFE) graft was used if a suitable autologous graft could not be obtained. Local application of heparinized saline during venous resections or a bolus dose of 5000 units of unfractionated heparin for arterial reconstructions were used. Postoperative anticoagulation with continuous heparin infusion was not implemented. Drains were placed in all patients and were removed between postoperative days 5 and 7 during an uneventful recovery. The clinical and surgical details are shown in *Table 1*.

**Table 1:** Demographic, clinical and treatment characteristics of extended pancreatic resections

Variable	Period A (*n* = 63)/range	Period B (*n* = 197)/range	*P*
Age (years), median (range)	54 (21–79)	57 (6–86)	0.512
**Sex**					
Male	36 (57.1)	115 (58.4)	0.521
Female	27 (42.9)	82 (41.6)	
BMI (kg/m^2^), median (range)	22.2 (13.5–31.25)	22.3 (12.2–39)	0.76
Albumin (g/dl), median (range)	3.89 (2.4–4.99)	3.93 (2.24–5.2)	0.27
**ASA grade**					
1	33 (52.4)	76 (38.6)	0.07*
2	27 (42.8)	100 (50.8)	
3	3 (4.7)	21 (10.7)	
**Diagnosis**					
Adenocarcinoma (pancreas)	27 (42.9)	107 (54.3)	0.095
Adenocarcinoma (ampulla of Vater)	3 (7.9)	10 (5.1)	
Adenocarcinoma (distal CBD)	5 (4.8)	5 (2.5)	
Adenocarcinoma (duodenum)	–	6 (3)	
Adenocarcinoma (stomach)	–	18 (9.1)	
Adenocarcinoma (colon)	6 (9.5)	3 (1.5)	
NET	5 (7.9)	21 (10.7)	
SPEN	5 (7.9)	13 (6.6)	
Other	9 (14.3)	14 (7.1)	
**Type of pancreatectomy**					
Pancreaticoduodenectomy	42 (66.7)	137 (69.5)	0.161
Distal pancreatectomy	19 (30.2)	51 (25.9)	
Subtotal pancreatectomy	1 (1.6)	–	
Total pancreatectomy	1 (1.6)	9 (4.6)	
**Type of pancreaticoduodenectomy**					
Pylorus-preserving	24 (57.1)	45 (32.8)	0.005*
Non–pylorus-preserving	18 (42.9)	92 (67.2)	
**Type of extended resection**					
Multivisceral only	27 (42.9)	64 (32.5)	0.178
Vascular only	31 (49.2)	111 (56.3)	
Combined multivisceral and vascular	5 (7.9)	22 (11.1)	
**Multivisceral resections†**					
Total	32 (50.8)	86 (43.6)	0.092
Single organ	22 (68.7)	75 (87.2)	
>1 organ	14 (43.8)	11 (12.8)	
**Additional organs resected**					
Total	32 (50.8)	86 (43.6)	0.003*
Colon	20 (62.5)	30 (34.9)	
Left adrenal gland	6 (18.6)	14 (16.3)	
Kidney	5 (15.6)	3 (3.5)	
Stomach	4 (12.5)	33 (38.4)	
Diaphragm	3 (9.4)	–	
Liver	2 (6.3)	6 (6.9)	
Small bowel	2 (6.3)	2 (2.3)	
Rectum	–	1 (1.2)	
Peritoneum	–	1 (1.2)	
Lymph nodes	–	6 (6.9)	
DJ flexure	–	1 (1.2)	
**Vascular resections†**					
Total	36 (57.1)	133 (67.5)	0.081
Vein resection	33 (91.7)	83 (62.4)	
Artery resection‡	1 (2.7)	9 (6.7)	
Vein + artery resection‡	2 (5.6)	13 (9.8)	
Artery divestment alone	–	14 (10.5)	
Vein resection + arterial divestment	–	14 (10.5)	
**Vein resections**					
Total	35 (97.2)	110 (80)	<0.001*
PV/SMV type I	7 (20)	24 (21.8)	
PV/SMV type II	–	4 (3.6)	
PV/SMV type III	20 (57.1)	74 (67.2)	
PV/SMV type IV	5 (14.3)	4 (3.6)	
PV/SMV-VROR	3 (8.6)	3 (2.7)	
IVC type II	–	1 (0.9)	
**Arterial resections§**					
Total	3 (8.3)	24 (17.7)	<0.001*
**Hepatic artery resection**					
End-to-end primary repair	1 (33.3)	5 (20.8)	
Interposition graft	2 (66.7)	3 (12.5)	
Coeliac axis resection (without reconstruction)	–	4 (16.6)
**Coeliac axis resection (with hepatic artery reconstruction)**	–	1 (4.2)	
End-to-end primary repair	–	5 (20.8)	
Interposition graft)	–		
**SMA**		1 (4.2)	
End-to-end primary repair	–	1 (4.2)	
Interposition graft	–	1 (4.2)	
Splenic artery (end-to-end primary repair)	–	2 (8.3)	
Left gastric artery (resection without reconstruction)	–	1 (4.2)	
Left gastric artery (end-to-end primary repair)			
**Arterial divestment#**			
Total	–	32 (24.4)	<0.001*
SMA	–	16 (51.5)	
CHA	–	10 (30.3)	
LGA	–	2 (6)	
RHA	–	1 (3)	
SA	–	1 (3)	
LHA	–	2 (6)	

Values are percentages unless otherwise stated. CBD, common bile duct; DJ, duodeno-jejunal; IVC, inferior vena cava; NET, neuroendocrine tumour; PV, portal vein; SMA, superior mesenteric artery; SMV, superior mesenteric vein; SPEN, solid pseudopapillary epithelial neoplasm; VROR, vein resection without reconstruction; CHA, common hepatic artery; LGA, left gastric artery; RHA, right hepatic artery; SA, splenic artery; LHA, left hepatic artery. *Statistically significant difference. †Includes patients undergoing combined multivisceral and vascular resection. ‡One patient in period B who underwent additional arterial divestment. §Two patients in period B underwent more than one artery resection. #Total of 32 arterial divestment procedures in 30 patients.

### Perioperative outcomes

There was no increase in the 90-day mortality (4 (6%) *versus* 12 (6.1%), *P* = 0.81), major morbidity (23 (36.5%) *versus* 83 (42.1%), *P* = 0.57) or failure-to-rescue rates (4 (17.4%) *versus* 12 (14.5%), *P* = 0.71) in period B, despite the increased complexity of surgical procedures. Median operative times were shorter in period B (510 min *versus* 420 min, *P* = 0.027), with a higher lymph-node yield (14 (2–38) *versus* 17 (1–80), *P* = 0.008) and with fewer patients requiring perioperative blood transfusions (35 (55.6%) *versus* 55 (41.3%), *P* = 0.018). Rates of DGE (grades B and C) were significantly higher (1 (1.6%) *versus* 21 (10.7%), *P* = 0.038) in period B.

Ideal outcomes were reached in 32 (50.7%) and 96 (48.7%) patients in periods A and B respectively (*P* = 0.776). Ideal outcomes were reached in 47 (51.6%), 62 (53.4%), 4 (40%), 4 (26.7%), 7 (50%) and 4 (28.6%) patients who underwent a multivisceral resection, vein resection, artery resection, combined artery–vein resection, arterial divestment and combined vein resection and arterial divestment respectively. The perioperative outcomes are shown in *Table 2.*

**Table 2:** Perioperative outcomes of extended pancreatic resections

Variable	Period A (*n* = 63)/range	Period B (*n* = 197)/range	*P*
Duration of surgery (min)	510 (235–645)	420 (120–820)	0.027*
Blood loss (ml)	1500 (400–23 000)	1500 (100–10 500)	0.563
Perioperative transfusion	35 (55.6)	55 (41.3)	0.018*
Transfusion units	2 (1–39)	1 (1–10)	0.285
**Hospital stay (overall)**	(13)	4–59 (15)	6–66 (0.885
MVR	14 (7–42)	12 (6–37)	0.46
VR	13 (5–59)	17 (7–61)	0.95
Combined	13 (4–48)	22 (12–66)	0.611
**90-day mortality (overall)**	4 (6)	12 (6.1)	0.81
MVR†	1 (3.7)	1 (1.56)	0.58
VR‡	2 (6.5)	7 (6.3)	0.9
Combined MVR + VR§	1 (20)	4 (18.1)	0.93
PDAC only¶	4 (14.8)	7 (6.5)	0.2
**90-day major morbidity (overall)**	23 (36.5)	83 (42.1)	0.57
MVR†	10 (37)	25 (39)	0.8
VR‡	11 (35.5)	46 (41.4)	0.56
Combined MVR + VR§	2 (40)	12 (54.5)	0.32
PDAC only¶	13 (48.1)	45 (42.1)	0.57
**Failure to rescue (overall)**	4 (17.4)	12 (14.5)	0.71
MVR†	1 (10)	1 (4)	0.57
VR‡	2 (18.2)	7 (15.2)	0.78
Combined MVR + VR§	1 (50)	4 (33.3)	0.71
PDAC only¶	4 (30.7)	7 (15.5)	0.25
**90-day mortality (vascular resection type)#**					
None	1 (3.7)	1 (1.56)	0.421
Vein resection	1 (3)	4 (4.8)	0.74
Artery resection§,**	1 (100)	1 (11.1)	0.2
Vein + artery resection**	1 (50)	3 (23.1)	0.53
Artery divestment alone	–	0 (0)	–
Vein resection + arterial divestment	–	3 (21.4)	–
**90-day major morbidity (vascular resection type)#**					
None	10 (37)	26 (40.6)	0.76
Vein resection	11 (33.3)	29 (34.9)	0.87
Artery resection§,**	1 (100)	5 (55.6)	0.6
Vein + artery resection**	1 (50)	9 (69.2)	0.66
Artery divestment alone	–	6 (42.8)	–
Vein resection + arterial divestment	–	8 (57.1)	–
**Ideal outcome reached**					
Overall	32 (50.8)	96 (48.7)	0.776
MVR	13 (48.1)	34 (53.1)	0.664
VR	18 (54.5)	44 (53)	0.881
Artery resection	0 (0)	4 (44.4)	0.389
Vein + artery resection	1 (50)	3 (23.1)	0.423
Artery divestment alone	–	7 (50)	–
VR + artery divestment	–	4 (28.6)	–
CR-POPF (B + C)	13 (20.6)	27 (13.7)	0.332
Bile leak	0 (0)	6 (3)	0.183
PPH (B + C)	3 (4.8)	7 (3.6)	0.823
DGE (B + C)	1 (1.6)	21 (10.7)	0.038*

MVR, multivisceral; VR, vascular; CR-POPF, clinically relevant postoperative pancreatic fistula; PPH, post-pancreatectomy haemorrhage; DGE, delayed gastric emptying. *Statistically significant difference. †Denominator comprising total multivisceral resections in the respective time period. ‡Denominator comprising total vascular resections in the respective time period. §Denominator comprising total combined resections in the respective time period. ¶Denominator comprising total number of each type of vascular resection in the respective time period. #Denominator comprising total number of pancreatic adenocarcinomas (PDAC) in the respective time period. **One patient in period B underwent additional arterial divestment.

### Factors affecting perioperative outcomes

Patient age (HR 1.116), preoperative albumin (HR 0.256), intraoperative blood loss (HR 1.0) and duration of surgery (HR 1.007) were identified as independent predictors of postoperative mortality. Cut-offs for predicting mortality were age > 58.5 years (AUC 0.723), preoperative albumin level < 3.81 g/dl (AUC 0.689), blood loss > 1900 ml (AUC 0.844) and duration of surgery > 532 min (AUC 0.747).

**Fig. 2:**
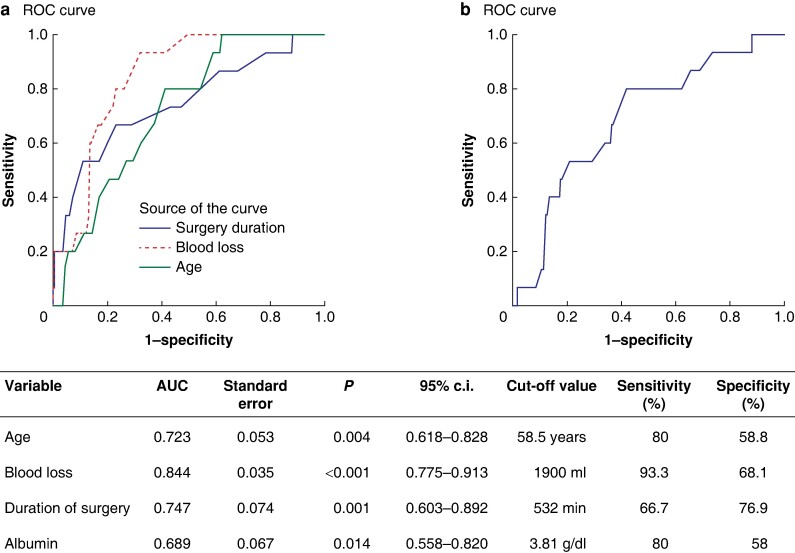
ROC curves of factors predicting postoperative mortality

Combined venous and multivisceral resections (HR 13.25), arterial resection or divestment procedures (HR 2.313), intraoperative blood loss (HR 1.00) and duration of surgery (HR 0.996) were identified as independent predictors of major morbidity. Regression output for perioperative outcomes was provided in *Table 3*, and ROC curves in *Fig. 2*.

**a** Patient age, intraoperative blood loss and duration of surgery; **b** preoperative albumin

**Table 3:** Logistic regression for factors affecting perioperative outcomes

Variable	Postoperative mortality				Postoperative major morbidity			
	Univariate *P*	Multivariate *P*	HR	95% c.i.	Univariate *P*	Multivariate *P*	HR	95% c.i.
Age (years)	0.008	0.01	1.116	1.1027,1.1212	0.349	–	–	–
Size (cm)	0.201	–	–	–	0.503	–	–	–
**ASA**								
1	Ref	–	–	–	Ref	–	–	–
2	0.730	–	–	–	0.371	–	–	–
3	0.130	–	–	–	0.595	–	–	–
BMI (kg/m^2^)	0.224	–	–	–	0.245	–	–	–
Preoperative biliary stent	0.057	–	–	–	0.843	–	–	–
Preoperative albumin (g/dl)	0.019	0.045	0.256	0.068,0.970	0.102	–	–	–
**Type of pancreatectomy**								
PD	Ref	–	–	–	Ref	–	–	–
DP	0.061	–	–	–	0.038	–	–	–
TP	0.503	–	–	–	0.279	–	–	–
**Complexity of surgery**								
Multivisceral	Ref	–	–	–	Ref	–	–	–
Vein resection	0.111	–	–	–	0.006	0.772	–	–
Multivisceral + vein resection	0.001	–	–	–	0.001	0.026	13.25	1.366,128.44
Arterial resection/divestment	0.002	–	–	–	0.003	0.029	2.313	1.090,4.911
**Additional organs resected**								
None	Ref	–	–	–	Ref	–	–	–
Single organ	0.452	–	–	–	0.518	–	–	–
>1 organ	0.255	–	–	–	0.183	–	–	–
Intraoperative blood loss (ml)	<0.001	0.001	1.000	1.000,1.001	<0.001	<0.001	1.000	1.000,1.001
Duration of surgery (min)	<0.001	0.006	1.007	1.002,1.012	0.997	0.005	0.996	0.994,0.999

DP, distal pancreatectomy; PD, pancreaticoduodenectomy; TP, total pancreatectomy.

### Outcomes of extended resections in pancreatic adenocarcinoma

A significantly greater number of BRPCs (5 (18.5%) *versus* 51 (47.7%), *P* = 0.011) and LAPCs (1 (3.7%) *versus* 24 (22.4%), *P* < 0.001) were operated in period B. NAT was more frequently used (6 (22.2%) *versus* 79 (73.8%), *P* < 0.001), with more prolonged chemotherapy (6 or more cycles in 5 (18.5%) *versus* 49 (45.8%), *P* < 0.001) in period B. The proportion of patients who received TNT was higher in period B, but the difference was not statistically significant (1 (3.7%) *versus* 19 (17.75%), *P* = 0.066).

An equivalent proportion of patients in both periods had PDAC (27 (42.9%) *versus* 107 (54.3%), *P* = 0.095). Overall node positivity rate in PDACs was significantly higher in period A (19/27 (70.4%) *versus* 46/107 (42.9%), *P* = 0.012). Node positivity rates among patients undergoing upfront surgery were similar in both periods (15/21 (71.4%) *versus* 19/28 (67.8%), *P* = 0.802). Node positivity rates among patients receiving NAT were similar between the two time periods (4/6 (66.7%) *versus* 27/79 (34.2%), *P* = 0.148). R-0 resection rates were similar in both periods (16 (59.3%) *versus* 65 (60.7%), *P* = 0.553), but vascular resections had a significantly higher rate of positive margins in period B (4 (6.3%) *versus* 24 (21.6%), *P* = 0.02) (*Table 4*).

**Table 4:** Clinicopathological characteristics of pancreatic ductal adenocarcinomas

Variable	Period A (*n* = 27)/range	Period B (*n* = 107)/range	*P*
Age (years), median (range)	60 (29–78	59 (27–86)	0.898
**Sex**					
Male	15 (55.6)	67 (62.6)	0.501
Female	12 (44.4)	40 (37.4)	
Baseline CA 19-9 (U/ml), median (range)	15.42 (2–36 800)	211.3 (1.4–48 757.2)	0.055
**Resectability†**					
Resectable	21 (77.8)	32 (29.9)	<0.001*
BRPC	5 (18.5)	51 (47.7)	
LAPC	1 (3.7)	24 (22.4)	
**Type of extended resection**					
Multivisceral only	10 (37)	14 (13.1)	0.02*
Vascular only	15 (55.5)	79 (73.8)	
Combined multivisceral and vascular	2 (7.4)	14 (13.1)	
**Vascular resections‡**					
Total	17 (62.9)	93 (86.9)	0.016*
Vein resection	15 (55.6)	53 (49.5)	
Artery resection§	1 (3.7)	8 (7.5)	
Vein + artery resection§	1 (3.7)	9 (8.4)	
Artery divestment alone	0	12 (11.2)	
Vein resection + arterial divestment	0	11 (10.3)	
**Neoadjuvant chemotherapy†**					
Yes	6 (22.2)	79 (73.8)	<0.001*
No	21 (77.8)	28 (26.2)	
Median neoadjuvant chemotherapy cycles	0 (0–12)	4 (0–14)	
**Neoadjuvant chemotherapy cycles†**					
None	21 (77.8)	28 (26.2)	<0.001*
<6	4 (14.8)	35 (32.7)	
6 or more	2 (7.4)	44 (41.1)	
**Neoadjuvant regimen†**					
None	21 (74.1)	28 (26.2)	<0.001*
FOLFIRINOX	2 (7.4)	64 (59.8)	
Gem-Nab-Paclitaxel	1 (3.7)	8 (7.5)	
Other	3 (11.1)	7 (6.5)	
Neoadjuvant radiation†	4 (14.8)	36 (33.6)	0.071
**Adjuvant therapy†**					
Yes	15 (55.6)	68 (54.4)	0.667
No	12 (44.4)	57 (45.6)	
**Tumour grade**					
Well-differentiated	0	4 (3.7)	0.018*
Moderately differentiated	16 (59.3)	78 (72.9)	
Poorly differentiated	11 (40.7)	25 (23.3)	
**pT stage**					
T1	1 (3.7)	17 (15.9)	0.061
T2	15 (55.6)	51 (47.7)	
T3	8 (29.6)	20 (18.7)	
T4	1 (3.7)	13 (12.1)	
CR	–	5 (4.7)	
LVI	16 (59.3)	39 (36.4)	0.225
PNI	18 (66.7)	55 (51.4)	0.031*
R0 rate	16 (59.3)	65 (60.7)	0.553
**Margin involved†**					
Transection	1 (3.7)	12 (11.2)	0.153
SMA/SMV	5 (18.5)	13 (12.1)	
Anterior/posterior surface	2 (7.4)	14 (13.1)	
Multiple	3 (11.1)	3 (2.8)	
**Nodal status**					
pN0	6 (22.2)	9 (8.4)	<0.001*
pN+	15 (55.6)	19 (17.6)	
ypN0	2 (7.4)	52 (48.6)	
ypN+	4 (14.8)	27 (25.2)	
Lymph node yield	10 (3–31)	17.5 (1–59)	0.013*
Adjacent organ invasion	11 (40.7)	50 (46.7)	<0.58*

BRPC, borderline resectable pancreatic cancer; LAPC, locally advanced pancreatic cancer; LVI, lymphovascular invasion; PNI, perineural invasion; SMA, superior mesenteric artery; SMV, superior mesenteric vein. *Statistically significant difference. †Includes patients of pancreatic adenocarcinoma only. ‡Includes patients undergoing combined multivisceral and vascular resection. §One patient in period B who underwent additional arterial divestment.

**Fig. 3:**
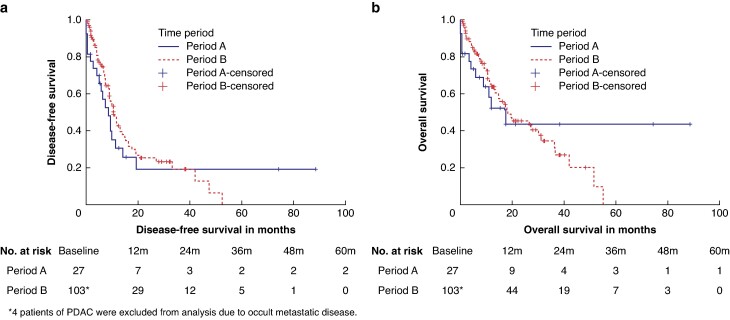
**Kaplan–Meier survival curves** for **a** disease-free survival and **b** overall survival of pancreatic ductal adenocarcinomas

Survival outcomes were analysed for PDAC only. The median duration of follow-up for PDAC was equivalent in both periods (12.6 (95% c.i. 7.45 to 17.78) *versus* 21.2 (15.67 to 27.7) months, *P* = 0.792). Overall, the median DFS of PDAC in both periods was equivalent (8.6 (95% c.i. 3.88 to 13.32 *versus* 10.67 (95% c.i. 8.52 to 12.82) months, *P* = 509) with an estimated 3-year DFS of 22.5% and 20.9% in periods A and B respectively. The median OS of PDAC in both periods was equivalent (17.5 (95% c.i. 6.77 to 28.22) *versus* 18.3 (95% c.i. 7.91 to 28.68) months, *P* = 0.958) with an estimated 3-year OS of 43.5% and 30.6% in periods A and B respectively. The survival curves of PDAC in period B are shown in *Fig. 3.*

None of the clinicopathological variables assessed was identified as an independent predictor of long-term survival on multivariate Cox regression. Details of the Cox regression have been elaborated in *Table S1*.

### Subgroup analysis

Survival outcomes of the subgroup of resectable PDACs were compared between the two time periods. All relevant clinicopathological characteristics were evenly matched between the two periods except lymph node yield, which was significantly higher in period B (median lymph node yield 11 *versus* 21 nodes, *P* = 0.045). Clinicopathological characteristics of the resectable PDAC subgroup have been summarized in *Table S2*.

**Fig. 4:**
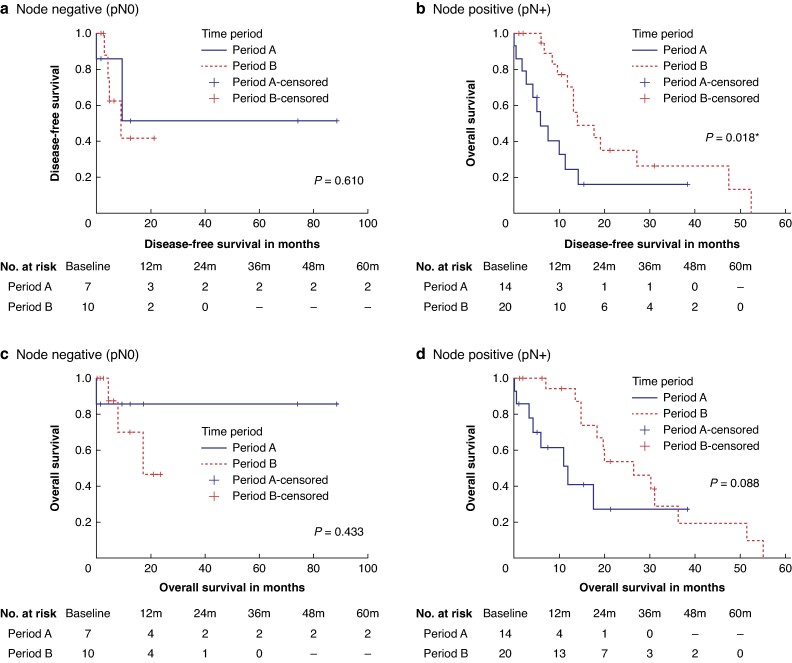
Subgroup analysis of resectable pancreatic adenocarcinoma

There was no difference in the DFS (median DFS 9.45 (95% c.i. 4.38 to 14.6) months *versus* 14.02 (95% c.i. 12.23 to 15.82) months, *P* = 0.370) or OS (median OS 17.5 months *versus* 26.5 months, *P* = 1.000) between the two periods. However, after stratifying for nodal status, the node-positive group had a significantly improved DFS in period B (median DFS 5.81 (95% c.i. 1.73 to 9.89) months *versus* 14.03 (95% c.i. 5.7 to 22.35) months, *P* = 0.018). Survival curves of these subgroup analyses are shown in *Fig. 4.*

Kaplan–Meier survival curves for **a** disease-free survival, node negative (pN0); **b** disease-free survival, node positive (pN+); **c** overall survival, node negative (pN0); and **d** overall survival, node positive (pN+)

The median DFS of isolated vein resections in period B was significantly longer than period A (9.49 (95% c.i. 6.24 to 12.75) *versus* 13.14 (95% c.i. 9.36 to 16.91) months, *P* = 0.028), with an estimated 2-year DFS of 0% and 30.5% in periods A and B, respectively. The survival curves are depicted in *Fig. S1*.

## Discussion

Significantly greater numbers of extended pancreatic resections were undertaken in period B, along with a foray into more complex arterial resection and divestment procedures and more frequent and prolonged use of NAT. Despite a significant increase in the complexity of surgery, surgical outcomes remained unchanged between the two periods. Four independent risk factors for perioperative mortality were identified: age, intraoperative blood loss, duration of surgery and preoperative albumin. Long-term survival remained unchanged between the two periods. A significantly longer DFS was observed in the subgroup of node-positive resectable pancreatic cancers, likely attributable to more radical surgery as evidenced by a higher lymph node yield in period B. DFS was significantly longer in the ‘vein resection only’ subgroup in period B as well.

The previous experience of extended pancreatic resections encompassed only a portion of the spectrum of extended resections, including only 35 (55.5%) portal vein resections and 3 (4.8%) arterial resections^21^. Despite the increased complexity of surgery in the present study, the surgical outcomes remained unchanged between the two periods and were comparable to similar series reported from high-volume pancreatic units worldwide^33–42^ (*Table S3*). Mortality and morbidity rates for extended resections in published surgical series range from 0% to 16.4% and 19.4% to 54.1%, respectively^33–41^. Truty *et al*.^33^ published a series of 194 extended resections, comprising 111 venous, 64 arterial, 50 combined and 38 multivisceral resections, with mortality and major morbidity rates of 6.7% and 35.6% respectively. Al Farai *et al*.^42^ have also published their results of 127 venous, 11 arterial and 32 multivisceral resections with mortality and morbidity rates of 6.2% and 60.8% respectively.

Augustinus *et al*.^25^ defined the criteria for ideal outcomes in pancreatic surgery in a sizeable transatlantic cohort study comprising 21 036 patients from North America, Germany, the Netherlands and Sweden. In their study, ideal outcomes were reached in 54% of patients. The results from the present analysis are comparable, with 128 (49.2%) patients reaching ideal outcomes. A closer look at the factors affecting perioperative outcomes identified four independent risk factors for perioperative mortality: age, intraoperative blood loss, duration of surgery and preoperative albumin. Patient age, an unmodifiable risk factor, should influence patient selection while considering patients for an extended pancreatic resection. Patient age of more than 58.5 years predicted mortality with a sensitivity and specificity of 80% and 58.8%, indicating a need for more stringent evaluation of patients > 60 years. This age cut-off may seem more conservative as far as pancreatic surgery is concerned. Although the safety and oncological benefit of a pancreatoduodenectomy has been demonstrated in a geriatric population^43^, one must emphasize that this benefit was restricted to patients with resectable periampullary malignancies undergoing standard pancreatic resections, as well as the fact that the current average life expectancy in India is only 70.4 years^44^. The remaining three factors, namely preoperative albumin, intraoperative blood loss and duration of surgery, are modifiable risk factors that serve to reiterate the core principles underlining improved surgical outcomes: careful patient selection, aggressive preoperative optimization, and meticulous surgical technique. Interestingly, the complexity of surgery was not independently associated with surgical mortality but only with morbidity, indicating that although increasingly complex resections are associated with higher complication rates, salvaging those complications in the postoperative period is the key to reducing the perioperative mortality rate.

In the earlier publication, extended pancreatic resections for PDAC were also associated with a significantly poorer DFS of 9.5 months as compared to 19.5 months with standard pancreatectomies, which was attributed to a high margin positive (R+) rate of 40.7% at the time^21^. Limited use of neoadjuvant therapy (26%) in the study also failed to reflect the current practice standard for advanced pancreatic cancer comprising more aggressive and prolonged neoadjuvant chemotherapy regimens. The median DFS and OS in period B were 11.72 months and 23.52 months respectively. Overall, the difference was not statistically significant between the two periods. However, despite similar R0 resection rates, a significantly longer DFS was observed in the subgroup of node-positive resectable pancreatic cancers, likely attributable to more radical surgery as evidenced by a higher lymph node yield in period B. DFS was significantly longer in the ‘vein resection only’ subgroup in period B as well. In period B, NAT was utilized more frequently with more radical resections, as indicated by more complex vascular resections and a significantly higher lymph node yield. A trend towards increased utilization of the TNT approach was also noted in period B. Increased utilization of neoadjuvant therapy as an effective strategy for management of advanced pancreatic cancers has been previously published by the authors^45^. Truty *et al*.^33^ have demonstrated improved survival with the use of six or more cycles of NACT in their series of extended pancreatic resections with a median DFS and OS of 23.5 months and 51.1 months respectively. The present study failed to identify any independent prognostic factors for survival on multivariate regression. The recently published PREOPANC trial has also shown a median OS of 14.6–15.6 for resectable PDACs and 13.2–17.6 months for BRPCs^46^. Other authors with similar series have reported median DFS and OS ranging from 7.4 to 14.9 months and 13.7–26.3 months, which are concurrent with the results from this study^33–42^ (*Table S3*).

A stepwise evolution of pancreatic surgery at the Tata Memorial Centre could be observed and correlated with the evolution of extended resections as well. Standardization of surgical technique^47^, establishment of a dedicated pancreatic unit^48^, implementation of the enhanced recovery after surgery (ERAS) protocols^49^, a concomitant increase in volumes^50^ and increased implementation of neoadjuvant strategies^45^ had contributed to the results in this study.

The present study had some limitations, such as the retrospective nature of the analysis. Elucidating factors affecting long-term outcomes remained challenging, given the heavy selection bias in allocating neoadjuvant treatment. Radiological assessment of resectability could vary between centres depending on the surgical and multidisciplinary expertise available at the concerned institute and did not entirely account for tumour biology. Assessment of nodal status on pathology could be affected by the downstaging effect of neoadjuvant therapy, the choice and duration of which depends on multiple factors such as tumour response, technical resectability, performance status, patient tolerance to chemotherapy and financial and social challenges. Accurately evaluating the treatment effect of currently employed neoadjuvant strategies would be best possible in a RCT design. The major strength of the study is that this was one of the largest reported series of extended pancreatic resections. It demonstrated the feasibility of more complex vascular resections with the same outcomes compared to series. The study also identified factors impacting perioperative outcomes that will enable accurate patient selection for complex resections.

Increasingly complex pancreatic resections were performed over time with consistent perioperative outcomes and improved DFS compared to the earlier period. A graduated approach to escalating surgical complexity, multimodality treatment and judicious patient selection enables resection of increasingly advanced pancreatic tumours. These results provided further justification for extended pancreatectomy in the era of modern chemotherapy for advanced pancreatic cancer.

## Supplementary Material

zrae065_Supplementary_Data

## Data Availability

The study participants did not give written consent for their data to be publicly shared; hence, supporting data are unavailable.
