# Supplementary material for: Evolution and improved outcomes in the era of multimodality treatment for extended pancreatectomy
Source: BJS Open. 2024 Aug 1;8(4):zrae065. doi: 10.1093/bjsopen/zrae065 (PMC11293468; doi:10.1093/bjsopen/zrae065)
Supplement: zrae065_Supplementary_Data [file zrae065_supplementary_data.docx]

**Title:** Extended pancreatectomy as defined by the ISGPS: Evolution and refined outcomes in the era of multimodality treatment.

**Authors**

1. *Vikram A Chaudhari^1^
2. *Aditya R Kunte^1^
3. Amit N Chopde^1^
4. Vikas Ostwal^2^
5. Anant Ramaswamy^2^
6. Reena Engineer^3^
7. Prabhat Bhargava^2^
8. Munita Bal^4^
9. Nitin Shetty^5^
10. Suyash Kulkarni^5^
11. Shraddha Patkar^1^
12. Manish S. Bhandare^1^
13. Shailesh V. Shrikhande^1^

***** Authors have contributed equally to the manuscript

**Author affiliations**

1. GI & HPB services, Department of Surgical Oncology, Tata Memorial Centre (Mumbai), Homi Bhabha National Institute.
2. GI & HPB services, Department of Medical Oncology, Tata Memorial Centre (Mumbai), Homi Bhabha National Institute.
3. GI & HPB services, Department of Radiation Oncology, Tata Memorial Centre (Mumbai), Homi Bhabha National Institute.
4. GI & HPB services, Department of Pathology, Tata Memorial Centre (Mumbai), Homi Bhabha National Institute.
5. GI & HPB services, Department of Interventional Radiology, Tata Memorial Centre (Mumbai), Homi Bhabha National Institute.

**Corresponding author.** Name and address

**Corresponding author**

Shailesh V. Shrikhande, MS, MD, FRCS (hon), FASA (hon).

Deputy Director, Tata Memorial Hospital

Professor and Chief of GI & HPB Oncology

Department of Surgical Oncology,

Tata Memorial Centre, Homi Bhabha National Institute.

Address: Department of Surgical Oncology, Tata Memorial Centre, Dr. Ernest Borges Road, Parel, Mumbai- 400012, India.

Email: [shailushrikhan](mailto:shailushrikhan)de@hotmail.com

Contact: +91 9820224761

**ORCID ID: 0000-0002-8036-4212**

**Supplementary Materials - Index**

| **Supplementary Methods** |  |
| --- | --- |
| - | *-* |
| - | *-* |
| **Supplementary Results** |  |
| - | *-* |
| - | *-* |
| **Supplementary Appendixes** |  |
| **-** | *-* |
| - | *-* |
| **Supplementary Figures and Tables** |  |
| Table S-1 | *Cox Regression for Factors Impacting Disease-free and Overall survival of Pancreatic Ductal Adenocarcinomas.*  *Cited on page 13, line 288 of the manuscript* |
| Table S-2 | *Clinicopathologic Characteristics of Resectable Pancreatic Adenocarcinoma subgroup*  *Cited on page 12, line 294 of the manuscript* |
| Table S-3 | *Literature Review of Extended Pancreatic Resections after 2017.*  *Cited on page 14, line 321 of the manuscript* |
| Figure S-1 | *Kaplan-Meier Survival Curves for Vein-only Resection subgroup* ***A)*** *Disease-free survival,* ***B)*** *Overall survival.*  *Cited on page 13, line 304 of the manuscript.* |
| **References** |  |
| - | *-* |

**Supplementary Figures and Tables**

**Supplementary Table S-1**. Cox Regression for Factors Impacting Disease-free and Overall survival of Pancreatic Ductal Adenocarcinomas.

| **Variable** | **Disease-free survival** | **Overall survival** |
| --- | --- | --- |
|  | **p- value** | **p- value** |
| **Age** | 0.767 | 0.629 |
| **Tumour size** | 0.736 | 0.748 |
| **CA 19-9** | 0.827 | 0.713 |
| **Resectability**  Resectable  BRPC  LAPC | Ref  0.541  0.377 | Ref  0.735  0.983 |
| **Number of NACT cycles** | 0.300 | 0.860 |
| **RT**  No  Yes | Ref  0.359 | Ref  0.952 |
| **Type of extended resection**  Multi-visceral (MVR)  Vein resection (VR)  MVR + VR  Arterial | Ref  0.255  0.231  0.251 | Ref  0.375  0.112  0.684 |
| **Type of pancreatic resection**  Left sided (DP/TP)  Right sided (PD) | Ref  0.,663 | Ref  0.901 |
| **Tumour differentiation**  WDAC  MDAC  PDAC | Ref  0.648  0.486 | Ref  0.884  0.824 |
| **Margin**  R0  R+ | Ref  0.997 | Ref  0.773 |
| **Lymph node yield** | 0.493 | 0.891 |
| **pN stage**  N0  N1  N2 | Ref  0.370  0.441 | Ref  0.906  0.457 |
| **LVI**  Absent  Present | Ref  0.233 | Ref  0.183 |
| **PNI**  Absent  Present | Ref  0.253 | Ref  0.593 |

* Statistically significant p-value

BRPC- Borderline resectable pancreatic cancer, LAPC- locally advanced pancreatic cancer, RT- Neo-adjuvant radiotherapy, DP- distal pancreatectomy, TP- total pancreatectomy, PD- pancreatoduodenectomy, WDAC- well differentiated adenocarcinoma, MDAC- moderately differentiated adenocarcinoma, PDAC- poorly differentiated adenocarcinoma, LVI- lymphovascular invasion, PNI- perineural invasion.

**Supplementary Table S-2.** Clinicopathologic Characteristics of Resectable Pancreatic Adenocarcinoma subgroup

| **Variable** | **Period A**  **(n = 21)** | ***%/ range*** | **Period B**  **(n = 32)** | ***%/ range*** | **p-value** |
| --- | --- | --- | --- | --- | --- |
| **Age** (median/range) | 57 | *29-78* | 60 | *30-76* | 0.536 |
| **Sex ratio (M:F)** | 13:8 | *61.9:38.1* | 22:10 | *68.8:31.3* | 0.607 |
| **Baseline CA 19-9** (median/range) | 96.67 | *2-221,711* | 198.32 | *2-16,405* | 0.572 |
| **Type of extended resection**  Multi-visceral only  Vascular only  Combined multi-visceral and vascular | 10  10  1 | *47.6*  *47.6*  *4.8* | 9  22  1 | *28.1*  *68.8*  *3.1* | 0.305 |
| **Vascular resections^∆^**  Total  Vein resection  Artery resection^+^  Vein + artery resection^+^  Artery divestment alone  Vein resection + arterial divestment | 10  9  1  1  -  - | *47.6*  *42.9*  *4.8*  *4.8*  *-*  *-* | 9  22  1  -  -  - | *28.1*  *68.8*  *3.1*  *-*  *-*  *-* | 0.220 |
| **Neoadjuvant chemotherapy (NACT)^#^**  Yes  No | 1  20 | *4.8*  *95.2* | 5  27 | *15.6*  *84.4* | 0.222 |
| **Neo-adjuvant regimen^#^**  None  FOLFIRINOX  Other | 20  -  1 | *95.2*  *-*  *4.8* | 27  3  2 | *84.4*  *9.4*  *6.3* | 0.157 |
| **Adjuvant therapy^#^**  Yes  No | 12  9 | *57.1*  *42.8* | 22  10 | *68.8*  *31.1* | 0.408 |
| **Tumour grade**  Well-differentiated  Moderately differentiated  Poorly differentiated | 1  13  7 | *4.8*  *61.9*  *33.3* | 1  21  10 | *3.1*  *65.6*  *31.2* | 0.726 |
| **pT stage**  T1  T2  T3  T4  CR | 1  12  8  -  - | *4.8*  *57.1*  *38.1*  *-*  *-* | 5  15  11  -  1 | *15.6*  *46.9*  *34.4*  *-*  *3.1* | 0.234 |
| **LVI** | 11 | *52.4* | 17 | *53.1* | 0.958 |
| **PNI** | 14 | *66.7* | 19 | *59.4* | 0.592 |
| **R0 rate** | 13 | *61.9* | 23 | *71.9* | 0.447 |
| **Nodal status**  N0  N+ | 7  14 | *33.3*  *66.7* | 11  21 | *34.4*  *65.6* | 0.938 |
| **Lymph node yield** | 11 | *3-31* | 21 | *2-59* | 0.045* |

**Supplementary Table S-3.** Literature Review of Extended Pancreatic Resections after 2017.

| **Author (year)** | **Pancreatectomy** | **Extended resection** | **Vascular resection** | **Peri-operative outcomes** | **Survival** |
| --- | --- | --- | --- | --- | --- |
| **Truty et al (2019)^33^** | Total- 25 (12.9%)  Subtotal- 47 (24.2%)  Whipple- 122 (62.9%) | Total 194  Venous- 111 (57.2%)  Arterial- 64 (33%)  Combined- 50 (25.8%)  Multi-visceral- 38 (19.6%) | HA- 37 (19.1%)  CA- 33 (17%)  SMA- 13 (6.7%)  Multiple- 16 (8.2%) | Mortality- 13 (6.7%)  Major morbidity- 69 (35.6%)  CR-POPF- 27 (13.9%)  PPH (B+C)- 22 (11.3%) | Median DFS- 23.5m  Median OS- 51.1m  3-year RFS- 32%  3-year OS- 59% |
| **Loveday et al (2018)^34^** | Total- 31  PD- 27  DP- 2  TP- 2 | Arterial- 20  Venous- 26 | SMA- 10  CA/HA- 10 | Mortality- 1 (5%)  Major morbidty- 6 (19.4%)  CR-POPF- 0%  PPH- 0% | Median OS- 25.9m  3- year OS- 28.1%  Median PFS- 14.9m |
| **Tee et al (2018)^35^** | Total- 111  PD- 45 (40.5%)  DP- 46 (41.5%)  TP- 20 (18%) | Arterial (planned)- 85  Arterial (unplanned)- 26  Concomitant venous- 57 (51.4%) | HA- 60  CA- 49  SMA- 15  Multiple- 15 | Mortality- 15 (13.5%)  Major morbidity- 60 (54.1%)  CR-POPF- 25 (22.5%)  PPH (B+C)- 17 (15.3%) | Median OS (upfront surgery)- 16.6m  Median OS (post NACT)- 53.6m |
| **Bacchelier et al (2018)^36^** | Total- 118  PD-51  DP- 49  TP- 18 | Artery- 118  Vein- 105 | SMA- 34  CA- 50  HA- 29 | Mortality- 6 (5%)  Major morbidity- 29 (24.6%)  CR-POPF- 7 (5.9%) | Median OS- 13.7m  3-year OS- 13%  Median DFS- 7.4m |
| **Yoshiya et al (2019)^37^** | n- 20- DP-CAR | Arterial- 20  Venous- 8 | Concomitant PV- 8 | Mortality- 0%  Major morbidity- 8 (40%)  CR-POPF- 8 (40%) | Median OS- 30.7m  5- year OS- 31.8%  5-year RFS- 9.8% |
| **Yamamoto et al (2017)^38^** | DP-CAR- 72 |  | PVR- 24 (33%) | Mortality- 3 (4.2%)  Major morbidity- 30 (42%)  CR-POPF- 23 (32%) | Median DFS- 9.3m  Median OS- 17.5m |
| **Klompmaker et al (2018)^39^** | 72 DP-CAR | Multivisceral- 28  Concomitant vein- 16 | SMA-1  CHA- 9 | Mortality- 11 (16.4%)  Major morbidity- 11 (16.4%)  CR-POPF- 14 (20.6%)  PPH- 6 (8.8%) | Median OS- 18m |
| **Torres et al (2019)^40^** | Total 72  PD- 49 (69%)  DP- 18 (25%)  TP- 5(7%) | Arterial – 13.8% | SMV- 25%  SMV-PV- 20/8% | Mortality- 13.9%  Morbidity- 48.7%  CR-POPF- 6 (8%) | Median OS (upfront)- 17.6m  Median OS (post- NACT)- 32.9m  Median DFS- 8.7m |
| **Rangelova et al.^41^** | Total 56  PD- 23 (44.2%)  DP- 5 (9.6%)  TP- 24 (46.2%) | Vascular resection- 42 (80.8%)  Venous- 39 (75%)  Arterial- 18 (34.6%)  Combined artery-vein- 15 (28.8%)  Multi-organ- 18 (34.6%) |  | Mortality- 3 (6%)  Morbidity-7 (28%)  CR-POPF- 4 (24%) | Median OS- 22.4m  3-year OS- 38.9% |
| **Farai et al (2019)^42^** | PD- 112 (86.2%)  DP- 16 (12.3%)  TP- 2 (1.5%) | Total 130  Venous (type 3)- 114  Venous (type 4)- 13  Arterial- 11 (8.5%)  Multi-visceral- 32 (24.6%) | CA- 6  SMA- 3  HA- 2 | Mortality- 8 (6.2%)  Major morbidity- 79 (60.8%)  CR-POPF- 18(13.8%)  PPH- 6 (4.6%)  DGE- 28 (21.5%) | Median OS- 26.3m  3-year OS- 38.5% |

**Figure S-1***.* Kaplan-Meier Survival Curves for Vein-only Resection subgroup **A)** Disease-free survival, **B)** Overall survival.

**
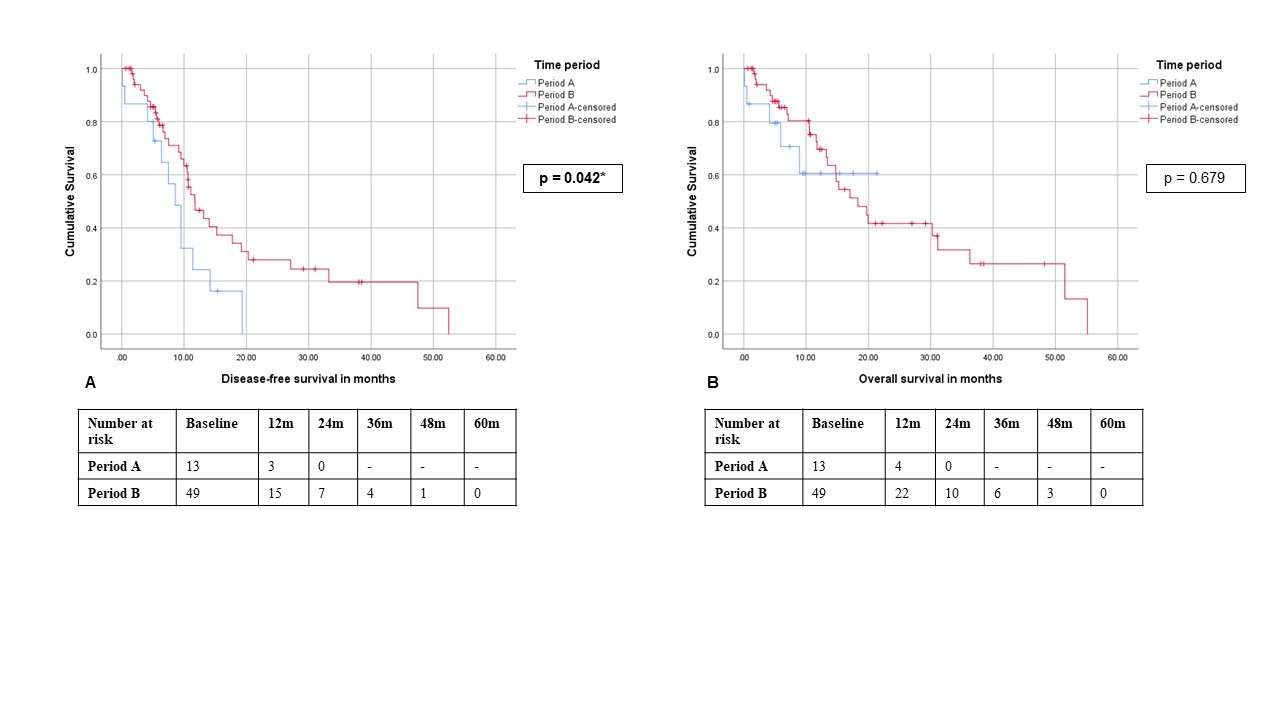
**
